# EP25 Blau syndrome: a lifetime of sarcoid

**DOI:** 10.1093/rap/rkaa052.024

**Published:** 2020-11-03

**Authors:** Neal Chauhan, Jessica Ellis, Sarah Emerson

**Affiliations:** r1 Rheumatology, North Bristol NHS Trust, Bristol, United Kingdom; r2 Rheumatology, Royal United Hospitals Bath NHS Foundation Trust, Bath, United Kingdom

## Abstract

**Case report - Introduction:**

Here we explore a case of a 31-year-old female treated with juvenile arthritis rebranded as Blau syndrome. This is characterised by sarcoid involvement principally of her eyes as well as skin, kidneys, and joints. We aim to explore her management challenges and complications.

**Case report - Case description:**

31 MS of Afro-Caribbean descent was diagnosed with early onset sarcoidosis at the age of 18 months. She was later found to be NOD2 gene positive but has no significant family history. Her health has been further complicated by her diagnoses of growth hormone deficiency and hyperthyroidism secondary to multinodular goitre.

Clinically MS has a short stature and profound deafness. A legacy of the ophthalmic morbidity, panuveitis, has led to a visual acuity is 6/15 in her left eye and her right reduced to counting fingers due to developing endophthalmitis in 2019. Her arthritis is stable, but there are flexion deformities primarily involving her proximal interphalangeal joints reducing her grip function. In adulthood she does not have significant cutaneous changes.

Investigations have revealed her to be anti-nuclear and anti-neutrophil cytoplasmic antibody negative. Radiologically, hand films show sparing of erosive changes, radiocarpal subluxation, and deformity of carpi in keeping with bilateral Madelung deformity. MRI has never suggested synovial enhancement of sacroiliac joints.

Therapeutically she has trialled conventional disease modifying therapy including azathioprine, mycophenolate, and methotrexate. Disease activity continues to be high despite anti-TNF biologic DMARDs (adalimumab, infliximab and golimumab). Use of anti-TNF therapy has been complicated by development of CMV disease and identification of anti-infliximab antibodies following interruption.

Ongoing steroid use from 18 months, hyperthyroidism and a chronic inflammatory state has led to osteoporosis. DEXA Z-scores reveal lumbar -2.7, right femoral neck -3.2 and left femoral neck -4.7. Systemic corticosteroid has tried to be offset through dexamethasone intravitreal implants.

Consequentially, uncontrolled disease burden has also led to significant input from orthopaedics, including bilateral total knee replacements by age 28.

However, with MS, we are hopeful with the commencement of anti-IL6 receptor therapy (Tocilizumab) that this may represent a new line of effective treatment.

**Case report - Discussion:**

Blau syndrome is a juvenile sarcoidosis characterised by the triad of granulomatous arthritis, recurrent uveitis, and dermatitis. It is inherited in an autosomal dominant pattern due to a missense mutation in the gene encoding for nucleotide-binding oligomerisation domain containing protein 2 (NOD2/CARD15). It is proposed that NOD2 as intracellular receptors, for antigen such as lipopolysaccharide, may subsequently act like toll-like receptors to cascade NF-kappa B and downstream inflammatory pathways. Its role is also implicated in other inflammatory conditions such as Crohn’s disease.

As represented in our case, uveitis presents the highest morbidity, with median age of eye disease presenting at 60 months and remains persistent despite topical or systemic therapy in more than 50% of patients. In comparison to Juvenile idiopathic arthritis, Blau often leads to a pan rather than anterior segment only uveitis and in a greater proportion of cases.

In addition to biological factors, much of MS’s treatment decisions have been guided by her and her psychology. In attempting to address the difficulties of her condition, including coordinating several specialities clinics whilst developing as a young woman, she has frequently discontinued therapy which may represent an area that she can exact control given little perceived benefit.

TNF-alpha inhibitors have been validated through randomised controlled trials for the use of refractory sarcoidosis. However recent literature has highlighted the use of targeting the IL-6 pathway in multisystem chronic sarcoidosis. It is believed this may represent a key cytokine in promoting Th17 effector cells that have been implicated in sarcoidosis bio-pathology. However ongoing case reports and ideally large-scale studies are needed.

**Case report - Key learning points:**

Blau syndrome as a rare lifelong form of early onset sarcoidosis offers insight into challenging therapeutic decision making where limited clinical trial data is available.

